# Population pharmacokinetic modeling of voriconazole based on real-world data for dosing regimen optimization

**DOI:** 10.3389/fphar.2026.1979373

**Published:** 2026-10-19

**Authors:** Li Jiang, Zhiqiang Lin

**Affiliations:** Department of Pharmacy, Quanzhou First Hospital Affiliated to Fujian Medical University, Quanzhou, Fujian, China

**Keywords:** dose adjustments, Monte Carlo simulation, population pharmacokinetics, Therapeutic drug monitoring, voriconazole

## Abstract

**Objectives:**

This study established a population pharmacokinetic model of voriconazole with the aim of adjusting the dosing regimen of voriconazole.

**Methods:**

This was a retrospective study in which clinical data from 3,002 patients were collected from their medical records. Based on the inclusion and exclusion criteria, this study included data from 96 patients to establish a population pharmacokinetic model. Following model evaluation, a dosing adjustment regimen for voriconazole was derived using Monte Carlo simulation and validated in a clinical setting.

**Results:**

The study used a one-compartment model to fit the data, with a exponential model selected for inter-individual variability and a mixed model selected for residual variability. The model results showed that the CL of voriconazole was 4.2 L/h and the V was 75.9 L. CL was significantly correlated with creatinine clearance (CCr), and C-reactive protein (CRP), while V was significantly correlated with whether omeprazole was used in combination. According to Monte Carlo simulation results: If the trough concentration is <0.5 mg/L, then the maintenance dose should be increased by 95%. If the trough concentration is ≥ 0.5 mg/L and <1.0 mg/L, then the maintenance dose should be increased by 65%. If the trough concentration is > 5.5 and ≤7.5 mg/L, then the maintenance dose should be decreased by 35%. If the trough concentration is > 7.5 and ≤10.0 mg/L, then the maintenance dose should be decreased by 55%. If the trough concentration is > 10.0 mg/L, then the maintenance dose should be decreased by 75%. In the clinical validation study, a total of 67 patients met the new inclusion and exclusion criteria and were included in the validation analysis. Of these 67 patients, after up to three dose adjustments based on the recommendations of the population pharmacokinetic model, 61 patients (91.04%) achieved steady-state plasma concentrations within the target range.

**Conclusion:**

67 patients participated in the clinical validation study. The results showed that among the 67 patients who followed the standard treatment regimen described in the package insert, 46.3% achieved the target concentration range. After applying the dose adjustment regimen derived from this study, the proportion of patients achieving the target concentration range increased to 91.04%.

## 1 Introduction

Fungal infections have remarkable morbidity and mortality worldwide, with approximately more than 13 million infections and 1.5 million deaths annually (McCreary et al., 2023). Although several novel antifungal drugs are already in development, triazoles remain the mainstay of therapy for the treatment and prevention of invasive fungal diseases. Voriconazole is a triazole antifungal agent with broad-spectrum activity, which is often recommended as the first regimen for invasive fungal infection (IFI) and as antifungal prophylaxis in immunocompromised patients. Therapeutic drug monitoring (TDM) is the measurement of drug concentrations at specified intervals to assess patient exposure and optimize individualized dosing programs. To improve the therapeutic efficacy and safety of voriconazole, the American and European Societies for Infectious Diseases recommend that voriconazole therapy should be routinely monitored for therapeutic agents (Gómez-López, 2020; Hope et al., 2012; Limper et al., 2011; Patterson et al., 2016; Ullmann et al., 2012). One study showed that approximately 46.0% of patients using the voriconazole dosing regimen recommended in the instructions had trough concentrations that were not within the target concentration range. In patients with concentrations outside the target range, dose adjustment increased the probability of attaining target trough concentrations by approximately threefold compared with no dose adjustment. (Lempers et al., 2019; Yi et al., 2017). Due to the highly variable pharmacokinetics of voriconazole, population pharmacokinetic models play an important role in optimizing dosing regimens and enabling individualized therapy (Liu et al., 2024; van den Born et al., 2023; Yang et al., 2021). Voriconazole guideline published by the Chinese Pharmacological Society recommend use of population pharmacokinetic models based on chinese populations for voriconazole dose adjustment (Chen et al., 2018). Voriconazole is extensively metabolized, with approximately 98% of the drug being eliminated via N-oxidation to form voriconazole N-oxide (Schulz et al., 2019). *CYP2C19* genotype-based dosing regimens represent a potential strategy for optimizing voriconazole administration. However, studies also indicate that poor metabolizers of *CYP2C19* (most commonly observed in Asian populations) may shift to alternative metabolic pathways for voriconazole, such as *CYP3A4* and *CYP2C9* metabolism pathways (Dean et al., 2012; Takesue et al., 2022; Zhang et al., 2021). Currently, none of the guidelines for voriconazole recommend routine pre-treatment genetic testing, but all recommend therapeutic drug monitoring after taking voriconazole (Chen et al., 2018; Takesue et al., 2022; Ashbee et al., 2014; Laverdiere et al., 2014; Chau et al., 2021). Additionally, studies indicate that without genetic testing, simply through trough monitoring, only two dose adjustments are required for 80% of patients to achieve the target concentration range (Perreault et al., 2019). Currently, most population pharmacokinetic studies of voriconazole consider *CYP2C19* as a influencing factor, and various dose adjustment regimens based on this genotype have been developed (Li et al., 2020; Zubiaur et al., 2021). However, although *CYP2C19* is a recognized determinant of voriconazole clearance, issues regarding clinical applicability make it difficult to apply the results of *CYP2C19*-based models to clinical practice. Therefore, the objective of this study is to develop a population pharmacokinetic model for voriconazole based on real-world clinical data. Based on the model results, a voriconazole dose adjustment regimen based on trough concentrations that is more suitable for clinical use will be established.

## 2 Methods

### 2.1 Study population

The investigators retrospectively collected real-world data from May 2018 to January 2025 on therapeutic drug monitoring after intravenous voriconazole administration in a tertiary care general hospital in China. This study has been reviewed and approved by the Ethics Committee of Quanzhou First Hospital. Specific inclusion and exclusion criteria were as follows:

The inclusion criteria for the study were as follows: (1) Patients with intravenous voriconazole (2) Adult patients aged ≥15 years or adolescent patients aged 12–14 years and body weight ≥50 kg, (3) At least two therapeutic drug monitors have been performed after application of voriconazole.

The exclusion criteria for this study were as follows: (1) The patient is taking medications that are contraindicated for use in combination with voriconazole, such as rifampin.

### 2.2 Information gathering

Patient information was obtained from the hospital’s electronic medical record system, and the initial dosing regimen was administered as directed (based on actual body weight, a loading dose of 6 mg/kg q12h iv was given, and a maintenance dose of 4 mg/kg q12h iv was given). The timing of blood collection is selected after steady-state concentrations are reached, and the timing of the first monitoring of trough concentration is generally selected before the 5th dose of the drug. The following information was recorded for each patient.

1. Demographic information: patient name, case number, age, body weight (WT), gender.
2. Laboratory data: white blood cell (WBC), neutrophils (N), platelets (PLT), total bilirubin (TBIL), interstitial bilirubin (IBIL), alanine aminotransferase (ALT), aspartate aminotransferase (AST), alkaline phosphatase (ALP), albumin (ALB), and C-reactive protein (CRP), blood urine nitrogen (BUN), serum creatinine (SCr), creatinine clearance (CCr), before and during drug administration.
3. Medication information: start and end of voriconazole administration, dosing regimen, co-administration (the three most frequently used concomitant medications that may potentially affect voriconazole metabolism), date and time point of blood collection, results of blood concentration tests.

### 2.3 Method for detecting voriconazole in plasma

The detection of voriconazole utilizes an enzyme multiplied immunoassay technique (EMIT). This method is based on the principle of a competitive immunoassay: Voriconazole in the sample competes with glucose-6-phosphate dehydrogenase (G6PDH) - labeled voriconazole for binding to specific antibodies. The concentration of voriconazole in serum is quantified by measuring the rate of NADH production (change in absorbance per minute) at a wavelength of 340 nm. The assay demonstrates a linear range of 0.5–16 μg/mL, with a linear correlation coefficient (r) of ≥0.9900. For concentrations ≤3 μg/mL, the absolute deviation from linearity is within ±0.3 μg/mL, while for concentrations >3 μg/mL, the relative deviation is within ±10%. The assay exhibits good precision, with a repeatability (coefficient of variation, CV) of ≤10% and an inter-assay CV of ≤15%.

### 2.4 Model development

The study used the nonlinear mixed effects modeling (NONMEM) method to develop a population pharmacokinetic model, and the principle of extended least squares was used to calculate the objective function value (OFV), which characterizes the deviation between the model simulated value and the real value, so the process of modeling is actually the process of seeking OFV minimization (Goutelle et al., 2022). Softwares used are PDx-POP (Version 5.3.1, companion graphical interface for NONMEM to execute, evaluate and visualise population PK/PD modelling and analysis) and R (Version 4.4.2).

#### 2.4.1 Development of base model

The commonly used structural models of voriconazole are the one - compartment model and the two - compartment model. Since the study only included trough concentration data, the two-compartment model exhibited significant fitting errors. Therefore, a one-compartment model was selected for fitting.

The between–subject variability (BSV) in pharmacokinetic parameters was estimated using an exponential function as follows:

$$P_{i}=\theta \times \exp\left(\eta_{i}\right)$$

Where Pi represents the estimated pharmacokinetic parameter for individual i, θ is the population typical value of the parameter, and η_i_ represents the random variable for individual i, which is defined as normally distributed with a mean of 0 and a variance of ω^2^.

The residual variability (RSV) was evaluated by additive, proportional, exponential, or combined residual error models. The equations were as follows:

$$\text{Additive error model }C_{\text{obs}}=C_{\text{pred}}+\varepsilon$$

$$\text{Proportional error model }C_{\text{obs}}=C_{\text{pred}}\times \left(1+\varepsilon \right)$$

$$\text{Exponential error model }C_{\text{obs}}=C_{\text{pred}}\times e^{\varepsilon }$$

$$\text{Combined error model }C_{\text{obs}}=C_{\text{pred}}\times \left(1+\varepsilon \right)+\varepsilon ’$$

In the above four equations, C_obs_ and C_pred_ are the observed and predicted concentrations, and ε and ε′ are random variables distributed with a mean of zero and a variance of ω^2^. The selection criteria were based on the decided structural model, further analyzing and comparing the OFV values and goodness-of-fit of the different random effects models, and selecting the best base model.

#### 2.4.2 Development of covariate model

The continuous variables age, WT, WBC, N, PLT, TBIL, IBIL, ALT, AST, ALP, ALB, CRP and the dichotomous variables methylprednisolone, omeprazole, and esomeprazole were studied separately. Missing data in the calculation process were imputed using the median. The specific process is as follows.

- Plot a covariate correlation plot for continuous variables, including WT, WBC, N, PLT, TBIL, IBIL, ALT, AST, ALP, ALB, and CRP.
- After analyzing the results, covariates with an absolute correlation coefficient |r| > 0.5 were considered to be highly correlated; a VIF >5 was considered to indicate moderate multicollinearity, while a VIF >10 was considered to indicate severe multicollinearity. Spearman’s rank correlation was used to analyze the correlations among continuous variables, and a heat map of the correlation matrix was plotted. Multiple linear regression diagnostics were also performed, and VIF values were calculated.
- The categorical variables are omeprazole, esomeprazole, and methylprednisolone. The comparison of categorical variables is conducted using chi square test or Fisher’s exact test, with P < 0.05 indicating statistical significance.
- Covariate selection was performed using a stepwise covariate modeling (SCM) approach consisting of forward inclusion followed by backward elimination. During forward inclusion, each candidate covariate was added to the base model one at a time; a covariate was retained if its inclusion resulted in a decrease in the OFV of more than 3.84 (P < 0.05). This step was repeated until no further covariate met the inclusion criterion, yielding the full model. In addition, for covariates with high correlation or multicollinearity, select the factor with the greatest decrease in OFV.
- During backward elimination, each covariate in the full model was removed one at a time; a covariate was retained in the model only if its removal increased the OFV by more than 6.63 (P < 0.01). The procedure was continued until all remaining covariates met the retention criterion, and the resulting model was defined as the final model.

### 2.5 Model evaluation

The adequacy of the fit was assessed by Goodness of fit plots (GOF) and Visual predictive check (VPC). The stability of the final model and the precision of the parameter estimates were assessed by resampling the raw data 10,000 times using the Bootstrap method and comparing the obtained parameters (95% CI and median) with the estimates of the final model.

### 2.6 Monte Carlo simulations

Monte Carlo simulation was performed using the final population pharmacokinetic model to derive dose adjustment recommendations. A virtual population of 4,000 subjects was generated, with covariate distributions resampled from the study population. The target steady-state trough concentration was defined as 1.0–5.5 mg/L. Based on historical clinical data, the simulation subjects were divided into six categories: <0.5, 0.5–1.0, 1.0–5.5, 5.5–7.5, 7.5–10, and >10 mg/L. For each categories, a range of dose modification factors (from −90% to +200% of the current maintenance dose, in 5% increments) was evaluated. Calculate the probability of reaching the target therapeutic concentration range of 1.0–5.5 mg/L (PTA) after up to three dose adjustments. The resulting values were rounded to the nearest clinically achievable dose increment (50 mg). Simulations were performed using [R, 4.4.2].

The formula for calculating trough concentration is as follows:

$$C_{\min}=\left(\text{dose}\times \text{BW }/\;V\right)\;*\;\left(\exp\left(-k\;*\text{ tau}\right)\;/\;\left(1-\exp\left(-k\;*\text{ tau}\right)\right)\right)$$

k=CL / V

where C_min_ is the trough concentration (mg/L), BW is body weight (kg), V is the volume of distribution (L), k is the elimination rate constant, CL is the clearance rate (L/h), and tau is the dosing interval (h).

### 2.7 Clinical validation of the model

To evaluate the predictive performance of the established population pharmacokinetic model for voriconazole, a clinical validation study was conducted to optimize and assess the proposed dosing regimen. Hospitalized patients who received intravenous voriconazole and underwent TDM between January 2025 and January 2026 were enrolled. To further assess the model’s utility in guiding individualized dosing in real-world clinical practice, additional exclusion criteria were applied during the model validation phase, beyond the predefined inclusion and exclusion criteria.

1. Patients who achieved the target therapeutic concentration after the initial dose.
2. Patients who, despite failing to reach the target concentration following the initial dose, did not receive dose adjustments according to model-recommended regimens or did not complete scheduled TDM after dose modification.

During this phase, dose adjustments were calculated by clinical pharmacists using the established voriconazole population pharmacokinetic model. Based on these model-informed recommendations, physicians modified the dosing regimens. Voriconazole concentrations were subsequently measured at steady state. The primary validation endpoint was the proportion of observed concentrations falling within the target therapeutic range (1.0–5.5 mg/L).

## 3 Results

### 3.1 Patient characteristics

This was a retrospective study in which clinical data from 3,002 patients were collected from their medical records. Based on the inclusion and exclusion criteria, data from 96 patients were included in the study. Ultimately, a total of 240 trough concentration records were collected for analysis of population pharmacokinetic data. The median (P25, P75) of the 240 trough concentration values was 3.7 mg/L (1.43, 6.68). Patients’ demographics and clinical characteristics are summarized in Table 1.

**TABLE 1:** Patient characteristics.

Characteristics	Median (P25, P75) N = 96
Male (N%)	74 (77.08%)
AGE (years)	64 (55, 74)
WT (kg)	55 (50, 61)
N (×10^9)	5.60 (2.66, 5.39)
CRP (mg/L)	48.86 (19.68, 80.18)
WBC (×10^9)	7.06 (3.98, 11.13)
ALT (U/L)	25 (18, 43)
AST (U/L)	34 (22, 53)
ALP (U/L)	102 (78,145)
ALB (g/L)	29.3 (27.5, 31.2)
PLT (×10^9)	152 (72, 261)
TBIL (μmol/L)	11.6 (9.03, 15.75)
IBIL (μmol/L)	6.9 (4.8, 9.3)
BUN (mmol/L)	5.97 (47.43, 71.00)
SCr (mmol/L)	58.3 (47.4, 71.0)
CCr (mL/min)	85.16 (62.27, 115.19)
Comedication	
Omeprazole (N%)	25 (26.04%)
Esomeprazole (N%)	19 (19.79%)
Methylprednisolone (N%)	32 (33.33%)

WT, body weight; WBC, white blood cell; N, neutrophils; PLT, platelets; TBIL, total bilirubin; IBIL, interstitial bilirubin; ALT, alanine aminotransferase; AST, aspartate aminotransferase; ALP, alkaline phosphatase; ALB, albumin; CRP, C-reactive protein; BUN, blood urine nitrogen; SCr, serum creatinine; CCr, creatinine clearance.

### 3.2 Collinearity

The covariance test for continuous variables shows that there is a high correlation between the following variables: N and WBC (r = 0.97), ALT and AST (r = 0.68), CCr and SCr (r = - 0.7), TBIL and IBIL (r = 0.81). A heatmap of specific covariate correlations is shown in Figure 1. Table 2 summarizes the results of collinearity diagnostics, indicating multicollinearity among several covariates. Specifically, N (tolerance = 0.103, VIF = 9.709), WBC (tolerance = 0.114, VIF = 8.744), TBIL (tolerance = 0.028, VIF = 35.822), and IBIL (tolerance = 0.029, VIF = 34.503) exhibited tolerance values <0.2 and VIF values >5, suggesting moderate to severe multicollinearity.

**FIGURE 1:**
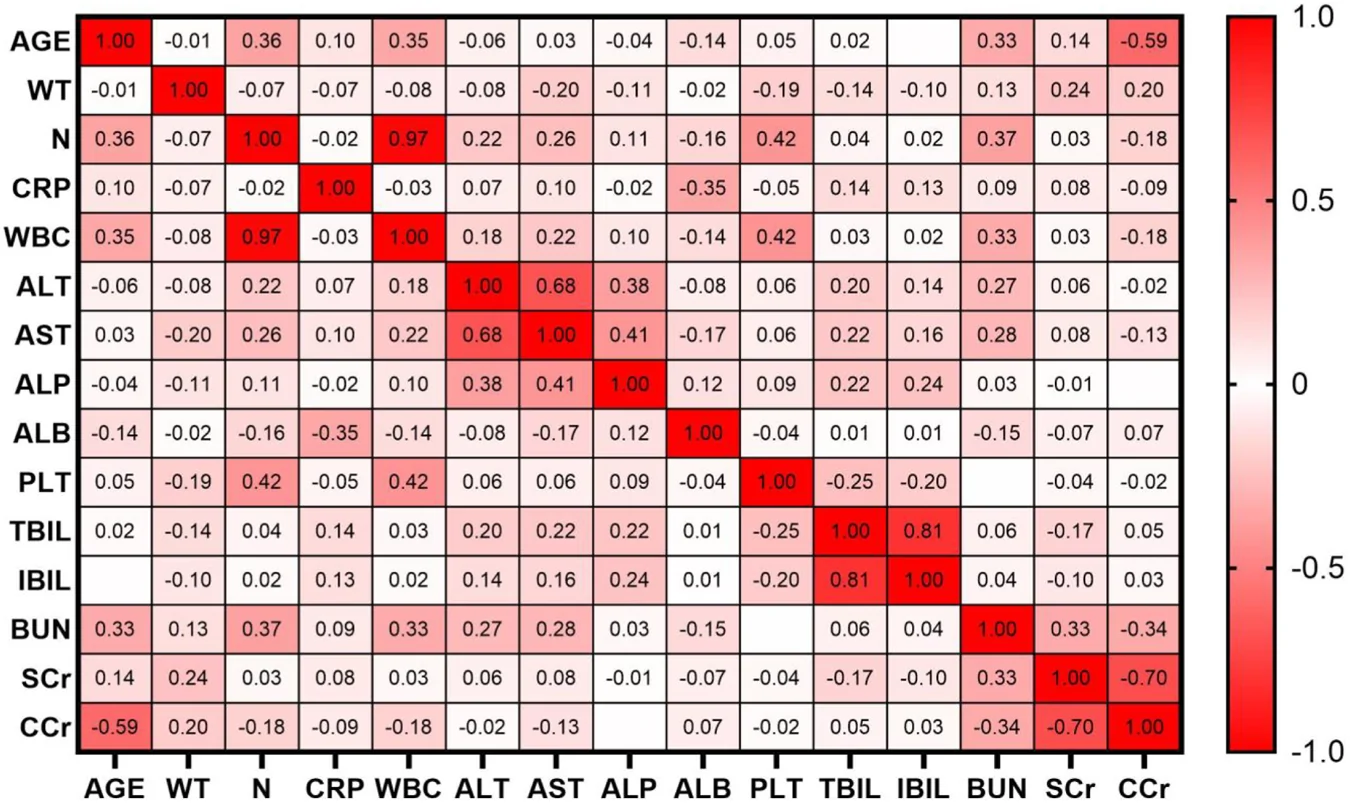
Covariance heatmap of covariates (the data in the heatmap represents the correlation coefficient r, and the blank indicates a value that is too small to be displayed. In the results, a positive or negative sign indicates a positive or negative correlation, respectively; a correlation coefficient with an absolute value |r| > 0.5 is considered to indicate a high correlation.).

**TABLE 2:** Diagnosis of multicollinearity in covariates.

Covariates	Tolerance	VIF
AGE	0.587	1.704
WT	0.747	1.338
N	0.103	9.709
CRP	0.885	1.130
WBC	0.114	8.744
ALT	0.286	3.498
AST	0.264	3.785
ALP	0.697	1.434
ALB	0.818	1.222
PLT	0.789	1.268
TBIL	0.028	35.822
IBIL	0.029	34.503
BUN	0.600	1.665
SCr	0.587	1.704
CCr	0.447	2.239

WT, body weight; WBC, white blood cell; N, neutrophils; PLT, platelets; TBIL, total bilirubin; IBIL, interstitial bilirubin; ALT, alanine aminotransferase; AST, aspartate aminotransferase; ALP, alkaline phosphatase; ALB, albumin; CRP, C-reactive protein; BUN, blood urine nitrogen; SCr, serum creatinine; CCr, creatinine clearance.

### 3.3 Model development

Only trough concentration values and not peak concentration values were available in the study data, the estimation of pharmacokinetic parameters was highly biased when evaluating the two-compartment model, and a one-compartment model was ultimately used to fit the data. Random effects models incorporate both between - subject variability and within - subject variability (WSV). After a comprehensive comparison of how well the models fit the data, it was found that the exponential model was chosen as the best fit for the BSV, and the combined (additive and proportional) model was chosen as the best fit for the RUV.

Correlations of voriconazole clearance and volume of distribution with covariates are shown in Figures 2, 3. When each covariate was tested individually using the base model, a series of covariates were retained that resulted in a decrease in the OFV (△OFV >3.84). These retained covariates are shown in Table 3. After the forward inclusion model–building step and the backward elimination model–building step, the final model was constructed, incorporating CCr and CRP as significant covariates for CL, and whether omeprazole was used as a significant covariate for V. The expression is as follows:

$$\text{CL }\left(L/h\right)=4.2\times {\left(\text{CCr}/87\right)}^{0.427}\times {\left(\text{CRP}/48.9\right)}^{-0.155}\times e^{\eta \text{CL}}$$

$$V\;\left(L\right)=75.9\times 1.79^{\left(\text{omeprazole}-1\right)}\times e^{\eta V}$$

**FIGURE 2:**
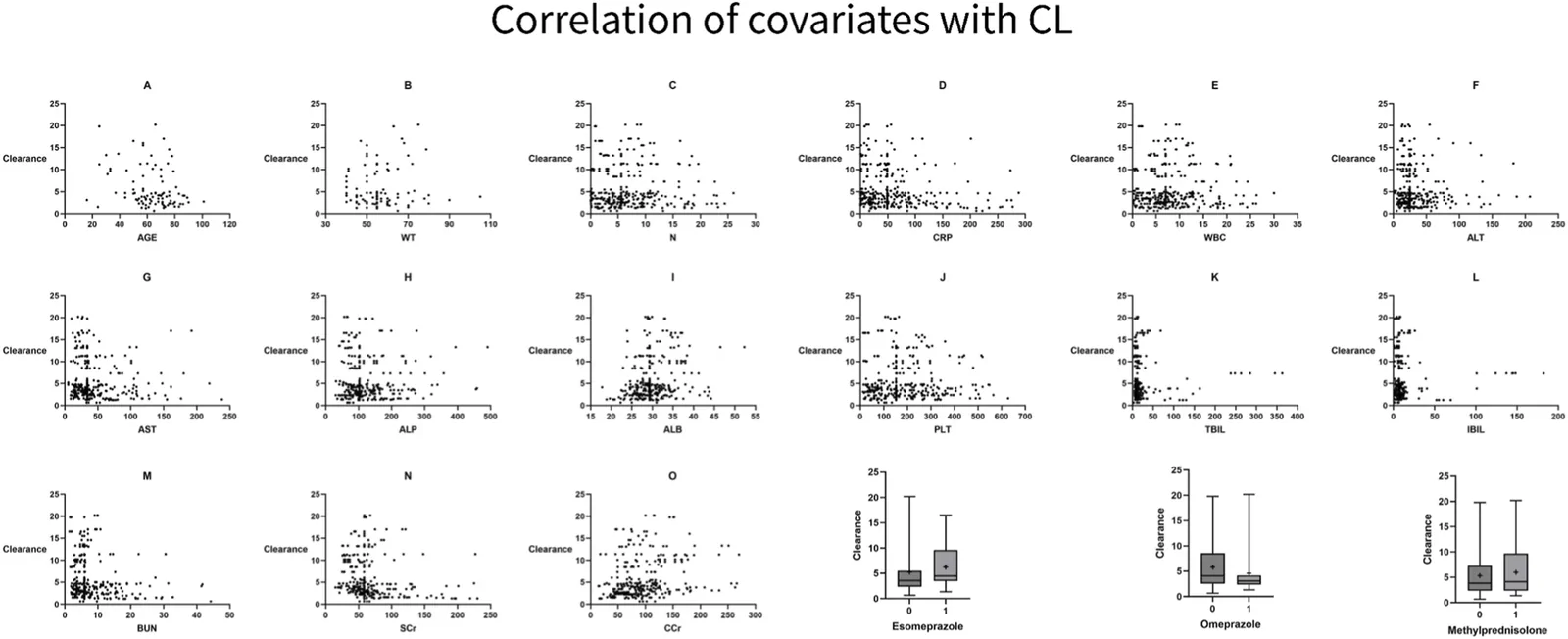
**(A)** Correlation of AGE with CL; **(B)** Correlation of WT with CL; **(C)** Correlation N with CL; **(D)** Correlation of CRP with CL; **(E)** Correlation of WBC with CL; **(F)** Correlation of ALT with CL; **(G)** Correlation of AST with CL; **(H)** Correlation of ALP with CL; **(I)** Correlation of ALB with CL; **(J)** Correlation of PLT with CL; **(K)** Correlation of TBIL with CL; **(L)** Correlation of IBIL with CL; **(M)** Correlation of BUN with CL; **(N)** Correlation of SCr with CL; **(O)** correlation of CCr with CL.

**FIGURE 3:**
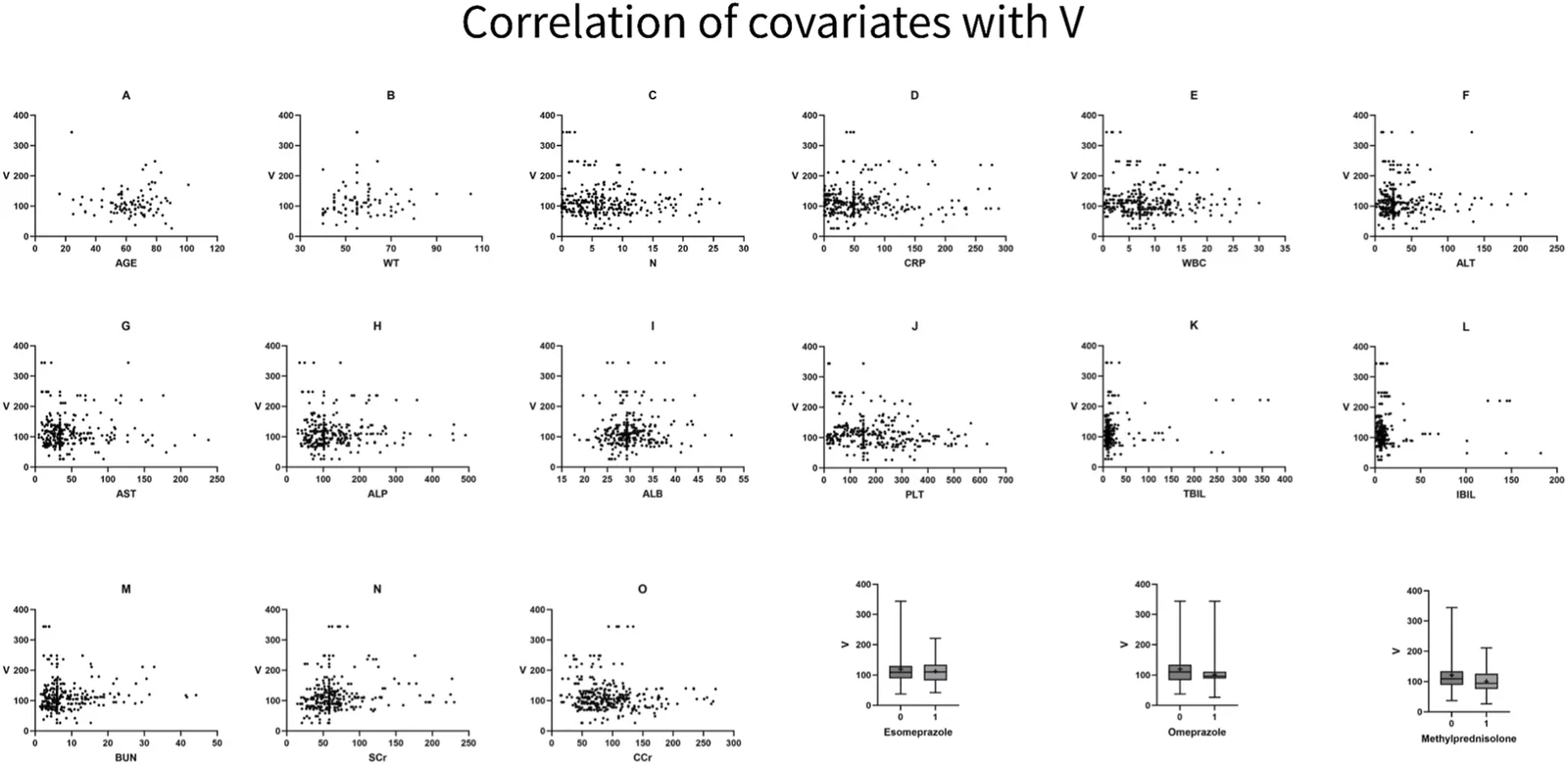
**(A)** Correlation of AGE with V; **(B)** Correlation of WT with V; **(C)** correlation N with V; **(D)** Correlation of CRP with V; **(E)** Correlation of WBC with V; **(F)** Correlation of ALT with V; **(G)** Correlation of AST with V; **(H)** Correlation of ALP with V; **(I)** Correlation of ALB with V; **(J)** Correlation of PLT with V; **(K)** Correlation of TBIL with V; **(L)** Correlation of IBIL with V; **(M)** Correlation of BUN with V; **(N)** Correlation of SCr with V; **(O)** Correlation of CCr with V.

**TABLE 3:** Results of screening of individual covariates with NONMEM.

Parameter	Significant covariate	△OFV	P Values
CL	AGE	−6.011	<0.05
​	CCr*	−12.884	<0.05
​	SCr*	−10.078	<0.05
​	CRP	−10.821	<0.05
​	Methylprednisolone	−6.404	<0.05
V	Omeorazol	−4.67	<0.05

CL, clearance; V, volume of distribution; CRP, C-reactive protein; SCr, serum creatinine; CCr, creatinine clearance.

* CCr, and SCr, exhibit collinearity; therefore, only the factor with the greatest variation in OFV, is selected for inclusion.

The pharmacokinetic parameters of the final model are shown in Table 4.

**TABLE 4:** Dose adjustment protocol.

Trough concentration	Dose adjustment	PTA
<0.5 mg/L	Maintenance dose should be increased by 95%	89.2%
0.5–1.0 mg/L	Maintenance dose should be increased by 65%	90.3%
5.5–7.5 mg/L	Maintenance dose should be decreased by 35%	97.9%
7.5–10 mg/L	Maintenance dose should be decreased by 55%	98.9%
>10 mg/L	Maintenance dose should be decreased by 75%	99.0%

The probability of reaching the target therapeutic concentration range (PTA).

### 3.4 Model evaluation

The OFV of the final model was reduced by 26.639 (△OFV = 721.056-694.417 = 26.639) compared to the base model, indicating that the inclusion of covariates helped to improve the model. GOF plots (Figure 4) showed that the final model significantly improved the accuracy and individualization of voriconazole concentration prediction through covariate screening and structural optimization. Bootstrap method based on data resampling technique was used for internal validation of the model. There were 9,644 successes in 10,000 datasets and final model convergence rate was 96.44%. The overall parameter estimates were close to the median value of the bootstrap analysis and within the 95% CI, suggesting that the final model was robust and accurate. Bootstrap results of the final model are shown in Table 5. The VPC results show that the median observation lies within the 90% prediction interval, and the prediction interval covers most of the observations, indicating that the model captures the variability of the data well (Figure 5).

**FIGURE 4:**
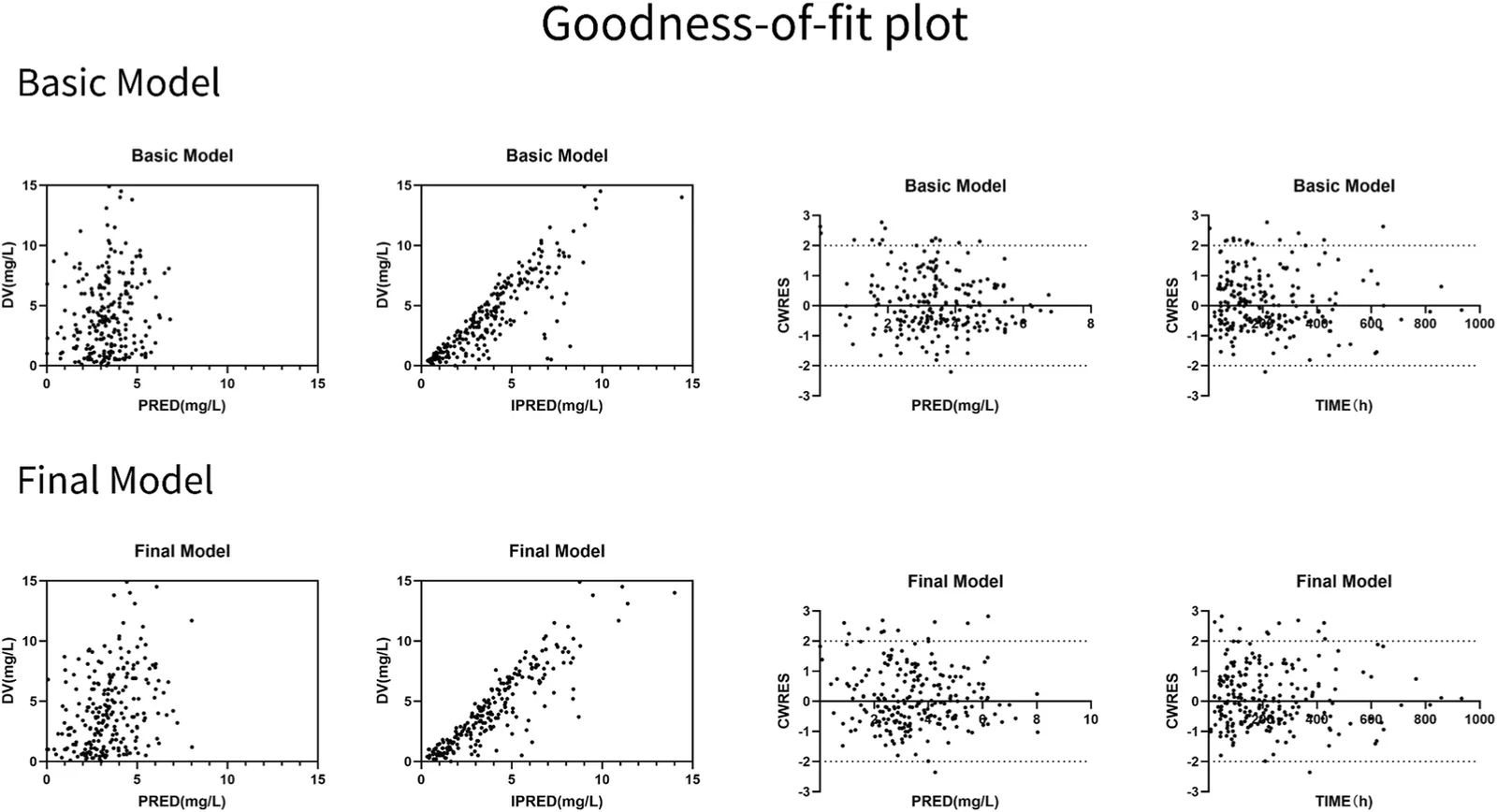
Goodness-of-fit plot.

**TABLE 5:** Model parameter.

Model parameter	Final Model (RSE%)	Bootstrap Median (95%CI)	Shrinkage (%)
OFV	694.287	681.5 (600.7,763.5)	—
CL (L/h)	4.2 (10.1%)	4.12 (3.17,5.10)	—
V(L)	75.9 (21.7%)	79.89 (45.80,119.00)	—
THETA_CCr_	0.427 (39.8%)	0.485 (0.156,0.993)	—
THETA_CRP_	0.155 (46.1%)	0.161 (0.003,0.311)	—
THETA_OMEP_	1.79 (23.5%)	1.961 (1.08,3.61)	—
OMEGA1(IIV-CL)	0.604 (18.0%)	0.573 (0.296,0.809)	η-shrinkage = 9.9%
OMEGA2(IIV-V)	0.393 (35.9%)	0.444 (0.014,1.770)	η-shrinkage = 58.7%
EPS(1)	0.169 (18.9%)	0.167 (0.099,0.236)	ε-shrinkage = 19.3
EPS(2)	0.247 (44.1%)	0.167 (0.099,0.236)	—

CL, clearance; V, volume of distribution; CCr, creatinine clearance; CRP, C-reactive protein; OMEP, omeprazole; RSErelative standard error; IIV, inter-individual variability.

**FIGURE 5:**
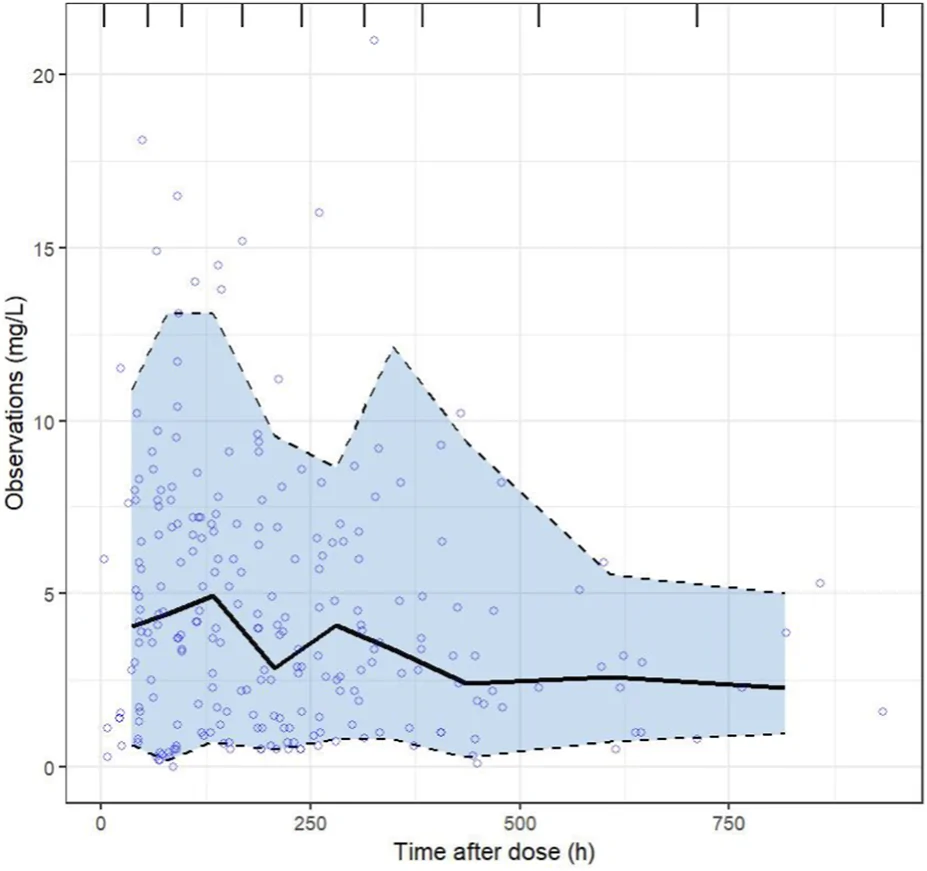
Visual prediction check chart (The solid black line represents the 50th percentile of the observed values, and the dashed black lines represent the 5th and 95th percentiles of the observed values).

### 3.5 Model-based simulation

In the final model, the covariates affecting CL were CRP and CCr, and the covariate affecting V was whether or not to co-administer omeprazole. Monte Carlo simulations were performed based on the model results to determine a dosing adjustment regimen for voriconazole. The specific results are shown in Table 4.

### 3.6 Clinical validation of the model

During the internal validation phase, 67 patients met the updated inclusion and exclusion criteria. The characteristics of the 67 patients are shown in Table 6. Following the standard dosing regimen recommended in the prescribing information, a total of 31 patients (46.3%) achieved the target concentration range. Following dose adjustments guided by the model, 61 patients (91.04%) achieved steady-state plasma concentrations within the target range.

**TABLE 6:** Patient characteristics in the clinical validation.

Characteristics	Median (P25, P75) N = 67
Male (N%)	46 (68.7%)
AGE (years)	62 (53, 80)
WT (kg)	55 (53, 65)
N (×10^9)	7.00 (5.29, 10.89)
CRP (mg/L)	52.31 (49.75, 85.00)
WBC (×10^9)	9.27 (6.96, 12.51)
ALT (U/L)	26 (16, 44)
AST (U/L)	27 (18, 41)
ALP (U/L)	84 (66,129)
ALB (g/L)	29.45 (26.15, 33.5)
PLT (×10^9)	127 (81, 258)
TBIL (μmol/L)	8.05 (6.55, 10.40)
IBIL (μmol/L)	6.15 (4.70, 7.10)
BUN (mmol/L)	8.46 (4.95, 12.1)
SCr (mmol/L)	64.15 (49.75, 85)
CCr (mL/min)	79.71 (58.35, 115.78)
Comedication	
Omeprazole (N%)	15 (22.40%)
Esomeprazole (N%)	18 (26.9%)
Methylprednisolone (N%)	10 (14.9%)

WT, body weight; WBC, white blood cell; N, neutrophils; PLT, platelets; TBIL, total bilirubin; IBIL, interstitial bilirubin; ALT, alanine aminotransferase; AST, aspartate aminotransferase; ALP, alkaline phosphatase; ALB, albumin; CRP, C-reactive protein; BUN, blood urine nitrogen; SCr, serum creatinine; CCr, creatinine clearance.

## 4 Discussion

### 4.1 PopPK model development

In this study, a single-compartment model of adult intravenous voriconazole population pharmacokinetics was developed based on real-world data. The exponential model was selected as the best-fitting model for BSV, while the combined (additive and proportional) model was selected as the best-fitting model for RUV.

The structural model used in most studies conducted in adults is the one-compartment model (Shi et al., 2019). This is because most studies employ trough concentration data, resulting in excessive errors during two-compartment model fitting (Mangal et al., 2018; Li et al., 2017). In addition, in most population pharmacokinetic studies, voriconazole is described as a linear process (Shi et al., 2019). There are studies that support this conclusion. Farkas' study compared the fitting of voriconazole concentrations by three models, linear, nonlinear, and mixed, and showed that simulations were most accurate using the linear model (Farkas et al., 2016). Therefore, in the present study, linear elimination was used to fit voriconazole with fewer pharmacokinetic parameters and is more applicable.

### 4.2 Model parameters

It has been shown that the median (range) of linear clearance of voriconazole in adult patients is 5.25 L/h (3.45–11.2) and the median (range) of the volume of distribution is 77.6 L (27.1–200) (Shi et al., 2019). In this study, the population estimate for V was 75.9, similar to the median. In contrast, the estimated CL was 4.2 L/h, which was lower than the reported median of 5.25 L/h but remained within the published range (3.45–11.2 L/h), supporting the plausibility of our findings. The observed reduction in CL may be attributed to the demographic characteristics of our cohort, in which the median (P25, P75) age was 64 (55, 74) years, indicating that the study population predominantly comprised older adults. Aging is associated with progressive renal senescence (Lin et al., 2022), characterized by a decline in glomerular filtration rate (GFR). It has been reported that GFR decreases by approximately 8 mL/min per decade after the age of 40 years, with an accelerated decline observed beyond 65–70 years of age (Lin et al., 2022; Wang J. et al., 2024). This age-related physiological decline may explain the slightly lower creatinine clearance observed in our study population; however, the exact causes and mechanisms remain to be investigated.

In addition, in this study, CCr was considered a factor influencing CL. Voriconazole is primarily metabolized hepatically and renal excretion of unchanged drug is minimal, the biological rationale for reduced renal function directly affecting voriconazole clearance is unclear. Regarding the impact of renal impairment on voriconazole plasma concentrations, studies have found that in kidney transplant recipients, the CL of voriconazole is indeed lower than in the general population (Lin et al., 2018). A large-scale PopPK study in critically ill patients provides strong evidence that CL is an independent factor influencing voriconazole clearance (Wang Y. et al., 2024). Although its mechanism of action requires further clarification, the clinical significance is clear: patients with better renal function may require higher doses or more frequent therapeutic drug monitoring to avoid subtherapeutic concentrations, while those with renal impairment must be vigilant against drug accumulation and toxicity risks (Wang Y. et al., 2024). Additionally, the intravenous formulation of voriconazole includes sulfobutylether beta-cyclodextrin (SBECD) as a solubilizer (Walsh et al., 2000). SBECD clearance is linearly related to creatinine clearance. It has been shown that hemodialysis patients have SBECD trough levels of 271–581 μg/ml after application of a voriconazole program of 200–400 mg q12 h iv, while patients with normal renal function have levels below 100 μg/ml. Although hemodialysis eliminates SBECD, SBECD trough levels in hemodialysis patients are still 6-fold higher than in patients with normal renal function (von Mach et al., 2006; Hafner et al., 2010). This indicates that long-term intravenous administration of voriconazole may also contribute to the deterioration of renal function. It has also been suggested that higher cumulative doses of intravenous voriconazole are a risk factor for renal dysfunction, and intravenous voriconazole should be avoided in patients with creatinine clearance <50 ml/min (Turner et al., 2015; Yasu et al., 2018).

It is worth noting that the range of V in this study is quite broad. This is because the study only included trough concentration data, making it difficult to estimate the value of the distribution volume. After conducting a literature search, it was determined that establishing the PopPK model for voriconazole using the NONMEM method is feasible (Lin et al., 2018; Chen et al., 2015; Li et al., 2021; Wang et al., 2014). Regarding this limitation, the primary objective of this study is to establish a population pharmacokinetic model for voriconazole suitable for clinical TDM practice, enabling the estimation of individual clearance rates. Although the model cannot precisely identify V, it reliably estimates CL and BSV based on trough concentration data, thereby facilitating the development of individualized dosing regimens. Therefore, the model is considered feasible.

### 4.3 Incorporation of covariates

Body weight, liver function and CYP2C19 genotype were common covariates affecting voriconazole in previous population pharmacokinetic studies (Shi et al., 2019). Other covariates were also examined in the modeling for this study; however, unfortunately, these covariates were not identified as significant predictors in the final model developed based on the current dataset. The dosing regimen for voriconazole in the instructions is based on body weight administration, suggesting that body weight may be a major source of pharmacokinetic variability. Han et al. conducted a population pharmacokinetic study in adult patients given 2 dosing regimens, either at a fixed dose or based on body weight, in order to investigate the extent to which the 2 dosing regimens reduced pharmacokinetic variability. The results showed that weight-based dosing did not reduce pharmacokinetic variability compared with the fixed-dose dosing regimen, and it was concluded that weight had no effect on the elimination of voriconazole (Han et al., 2010). However, there are also current studies that suggest a more appropriate voriconazole dosing strategy based on ideal body weight (IBW) or adjusted body weight (ABW) (Diller et al., 2021). In the present study, actual body weight was used for model development, which may explain why body weight was not identified as a significant covariate influencing voriconazole pharmacokinetics. Future studies should consider employing alternative weight metrics, such as ideal body weight or adjusted body weight, to evaluate whether different weight estimation methods exert a significant effect on voriconazole trough concentrations.

Voriconazole is metabolized by the liver, and some studies have found that voriconazole blood levels are related to ALT, AST, ALP, and bilirubin levels (Chen et al., 2015; Muto et al., 2015; Ren et al., 2019). In the present study, common monitoring parameters of liver function including ALT, AST, ALP, TBIL, IBIL had no effect on the pharmacokinetic parameters of voriconazole, which is not in accordance with the above study. These findings may be attributed to differences in the study population compared with previous investigations. The present cohort predominantly comprised older adults, in whom hepatic function is generally declined relative to younger populations. Moreover, given the real-world clinical nature of this study, the influence of hepatic function on voriconazole metabolism may have been confounded or masked by other more dominant covariates, such as underlying disease status. At the same time, due to practical limitations in this study, it was difficult to obtain patients’ Child-Pugh scores, making it impossible to comprehensively evaluate their liver function; this may also be one of the reasons, and it is an area for improvement in our future research.

In this study, the factors affecting the pharmacokinetic parameters of voriconazole were CRP, CCr and omeprazole. Numerous studies have reported that inflammation, which can be reflected by CRP levels, may influence voriconazole trough concentration (Veringa et al., 2017; Vreugdenhil et al., 2018; Gautier-Veyret et al., 2019). The results of one study showed that for every 1 mg/L increase in CRP values, voriconazole trough concentration increased by 0.015 mg/L (van Wanrooy et al., 2014). Studies have shown that drug metabolizing enzymes, including cytochrome P450 (*CYP*) isoenzymes, are reduced at the transcriptional level during infection or inflammation, resulting in decreased metabolism of voriconazole (Veringa et al., 2017). A significant negative correlation between C-reactive protein values and the metabolic rate of voriconazole suggests that the inflammatory state may play an important role in the high variability of voriconazole pharmacokinetics (van Wanrooy et al., 2014). In this study, the results for CL can be expressed using the following formula:

$$\text{CL }\left(L/h\right)=4.2\times {\left(\text{CCr}/87\right)}^{0.427}\times \exp\left[-0.155\;\ln\left(\text{CRP}/48.9\right)\right]$$

It can be seen that, when CCr remains constant, the slope of ln (CL) versus ln (CRP) is −0.155. This indicates that for every twofold increase in CRP, CL becomes 0.90 times its original value—that is, it decreases by about 10%. This is consistent with the direction of other studies. At the same time, CRP may partially overlap with the function of hepatic metabolic enzymes, thereby masking the effects of liver function. This may also explain why liver function markers were not included.

Voriconazole is metabolized by enzymes that primarily include *CYP2C19, CYP3A4*, and *CYP2C9*, and theoretically, the concomitant use of inducers or inhibitors of these drug-metabolizing enzymes should affect the pharmacokinetics of voriconazole (Hyland et al., 2003; Theuretzbacher et al., 2006). Administration of voriconazole with proton pump inhibitors (PPIs) and glucocorticoids is common in clinical practice. The effect of PPIs and glucocorticoids on voriconazole pharmacokinetics remains controversial, with most studies suggesting that it is related to the dose of the interacting drug (Dolton et al., 2012; Cojutti et al., 2016; Qi et al., 2017). The three most frequently co-prescribed concomitant medications in this study were omeprazole, esomeprazole, and methylprednisolone. Cojutti et al. demonstrated that PPIs affect voriconazole trough concentration to varying degrees, with 80 mg omeprazole exerting a greater effect than 40 mg omeprazole (Cojutti et al., 2016). Concomitant glucocorticoid use has also been shown to significantly increase voriconazole clearance (Huygens et al., 2023), indicating that both PPIs and glucocorticoids can meaningfully influence voriconazole exposure. In this study, a total of 76/96 (79.2%) patients were treated with drugs that affect the trough concentration of voriconazole, but only omeprazole had a significant effect on the trough concentration of voriconazole. This indicates that, in the data from this study, methylprednisolone had a smaller effect on the trough concentration of voriconazole than PPIs, and among the PPIs, omeprazole had a greater effect than esomeprazole. Further detailed research is needed to investigate the effects of these two common drugs on the trough concentration of voriconazole.

Recent studies have investigated the potential relationship between genetic variants such as *CYP2C19, CYP3A4, ABCB1, ABCC2, FMO3*, and *POR* and the pharmacokinetics and pharmacodynamics of voriconazole (Li et al., 2023). Among these genes, *CYP2C19* has the strongest effect on voriconazole metabolism and clearance, followed by *CYP3A4* (Li et al., 2023). The *ABCB1* and *ABCC2* genotypes may affect the transport and distribution of voriconazole, while the *FMO3* and *POR* genotypes may affect its metabolism and clearance (Allegra et al., 2018; Le et al., 2020; Zeng et al., 2020). The *CYP2C19* gene polymorphism was the most influential, accounting for approximately 50% of the voriconazole variability (Amsden and Gubbins, 2017). Therefore, polymorphisms in the *CYP2C19* gene, which encodes the *CYP2C19* isoenzyme, may be a major source of pharmacokinetic variability in voriconazole. Currently, most studies retained the *CYP2C19* genotype as an important covariate in the final model, and in only a few studies was the *CYP2C19* genotype considered to have no effect on voriconazole pharmacokinetic parameters (Jiang et al., 2022). In addition, there are a large number of confounding factors and a lack of consistent findings in these studies. Currently, there are no guidelines recommending that clinical administration of voriconazole should be routinely genetically tested, and all recommended interindividual doses are based on trough concentrations of voriconazole. The reasons for this may be as follows: 1) Poor C*YP2C19* metabolizers, most commonly in Asian populations, may switch to other metabolic pathways of voriconazole, such as *CYP3A4* and *CYP2C9* metabolic pathways (McCreary et al., 2023). The role of *CYP3A4* was enhanced in *CYP2C19* weak metabolizers (Poor metabolizers, PM) (Zhong et al., 2018). This means that monitoring only a single *CYP2C19* genotype may be insufficient. 2) Clinical practice has many factors that affect *CYP2C19*. Increased CRP concentrations significantly reduced voriconazole CL in patients with *CYP2C19* normal metabolite (NM) and intermediate metabolite (IM) (Ling et al., 2024). *CYP2C19* genotypes affects voriconazole exposure is age dependent. Influence of *CYP2C19* poor metabolizer genotype on increased voriconazole exposure is prominent in the young, while age is a more important determinant factor for increased voriconazole exposure in the elderly patients (Du et al., 2024). It has also been suggested that *CYP2C19* genotype has no effect on trough concentration in patients with severe hepatic impairment (Zhao Y. et al., 2021; Zhao Y. C. et al., 2021; Jiang and Lin, 2024). This means that in clinical practice, the effect of genotype may be masked by other factors. 3) Routine genetic testing prior to treatment can lead to higher costs. Current guidelines for voriconazole all recommend routine therapeutic drug monitoring and dose adjustments based on trough concentration. One study showed that 55% of patients whose trough concentration was not in the therapeutic concentration range were dose-adjusted, and 82% of these patients were able to adjust their trough concentration to the therapeutic concentration range (Lempers et al., 2019). This suggests that dose adjustment alone without genetic testing is a clinically feasible option. Based on the above three reasons, it is currently considered feasible to adjust dosing solely based on trough concentration indicators without considering *CYP2C19* genotypes. This is also why this study chose to explore the establishment of a voriconazole population pharmacokinetic model without incorporating *CYP2C19* genotypes.

During the internal clinical validation phase, 6 patients (8.96%) failed to achieve voriconazole trough concentrations within the target therapeutic range. Of these, 3 patients underwent a single dose adjustment but remained outside the target range; however, they were transferred to other hospitals due to worsening of underlying diseases, precluding further therapeutic drug monitoring and dose optimization. The remaining 3 patients persisted below the target range despite dose adjustment and were ultimately switched to alternative antimicrobial agents.

In addition to the uncertainty surrounding the precise estimation of the volume of distribution, several other limitations should be acknowledged. First, the sparse sampling schedule and the exclusive use of trough concentration data limited the accurate estimation of certain pharmacokinetic parameters. Second, voriconazole is primarily metabolized in the liver, and hepatic function is known to influence its trough concentrations. Since routine coagulation monitoring was not performed, the Child–Pugh score could not be incorporated as a covariate; instead, surrogate markers of hepatic function were used. This approach may have led to an underestimation of hepatic contributions in the model. Furthermore, CYP2C19 genotyping was not conducted, precluding a quantitative assessment of its specific impact on voriconazole pharmacokinetics. Third, while this study confirmed the effectiveness of the model-informed dosing regimen, its safety profile remains to be evaluated in future prospective studies. Although strict adherence to the model yielded a high target attainment rate, its generalizability in complex, real-world clinical settings requires further investigation. Fourth, the study population was predominantly composed of older adults, and the limited demographic and clinical diversity may have introduced bias into the results. These limitations likely explain why 6 of 67 patients (8.96%) failed to achieve the target concentration range despite model-guided dose adjustments. Additionally, the model was restricted to patients receiving intravenous voriconazole. The exclusion of oral administrations was primarily due to the retrospective design of the study, which made it difficult to control for confounding factors such as diet and to accurately ascertain dosing times. Consequently, the model’s applicability to orally dosed patients warrants further exploration in future research.

## 5 Conclusion

The OFV of the final model was reduced by 26.639 compared to the base model, indicating that the inclusion of covariates helped to improve the model. At the same time, internal validation has shown that it exhibits good stability and predictive performance. The model results indicate that factors affecting the trough concentration of voriconazole include CCr, CRP, and concomitant use of omeprazole. Using this model, a voriconazole dose adjustment regimen based on trough concentrations was developed. Clinical validation of the model showed that, among the 67 patients who followed the standard treatment regimen described in the package insert, 46.3% achieved the target concentration range. After applying the dose adjustment regimen derived from this study, the proportion of patients achieving the target concentration range increased to 91.04%.

## Data Availability

The original contributions presented in the study are included in the article/Supplementary Material, further inquiries can be directed to the corresponding author.

## Supplementary material

SUPPLEMENTAL TABLE S1 Model development.

## Glossary

ABW: adjusted body weight
ALB: albumin
ALP: alkaline phosphatase
ALT: alanine aminotransferase
AUC: Area Under Curve
AST: aspartate aminotransferase
BSV: between – subject variability
BUN: blood urine nitrogen
CL: clearance
CV: coefficient of variation
CRP: C-reactive protein
CCr: creatinine clearance
CYP450: Cytochrome P450
EMIT: enzyme multiplied immunoassay technique
G6PDH: glucose-6-phosphate dehydrogenase
GOF: goodness of fit plots
IBW: ideal body weight
IBIL: interstitial bilirubin
IM: intermediate metabolite
IFI: invasive fungal infection
MIC: Minimum Inhibitory Concentration
N: neutrophils
NONMEM: nonlinear mixed effects modeling
NM: normal metabolite
OFV: objective function value
PK/PD: Pharmacokinetics/pharmacodynamics
PLT: platelets
PM: Poor metabolizers
PTA: probability of achieving the target
PPIs: proton pump inhibitors
r: linear correlation coefficient
RSV: residual variability
SBECD: sulfobutylether beta-cyclodextrinm
SCr: serum creatinine
SCM: stepwise covariate modeling
TBIL: total bilirubin
TDM: therapeutic drug monitoring
V: volume
VPC: visual predictive check
WT: Weight
WBC: white blood cell
WSV: within - subject variability
